# Physiotherapists’ knowledge of and adherence to evidence-based practice guidelines and recommendations for ankle sprains management: a cross-sectional study

**DOI:** 10.1186/s12891-022-05914-5

**Published:** 2022-11-11

**Authors:** Giulia Caffini, Simone Battista, Andrea Raschi, Marco Testa

**Affiliations:** Department of Neurosciences, Rehabilitation, Ophthalmology, Genetics, Maternal and Child Health, University of Genova, Campus of Savona, via Magliotto 2 –, 17100 Savona, Italy

**Keywords:** Ankle injuries, Ankle joint, Ankle fractures, Physical therapy modalities, Physical therapists, Rehabilitation, Practice guidelines as topic

## Abstract

**Background:**

Lateral ankle sprain (LAS) is a common and burdensome injury. However, the quality of its management is scant. Nowadays, physiotherapy management of musculoskeletal diseases seems to be generally not based on research evidence. Studies that investigated the knowledge-to-practice gap in LAS management are yet to be carried out. Therefore, this research investigated physiotherapists’ knowledge of and adherence to LAS Clinical Practice Guidelines (CPGs) and recommendations.

**Methods:**

A cross-sectional study based on an online survey structured in three sections. The first section collected demographic data. The second section showed two clinical cases (with positive and negative Ottawa Ankle Rules (OAR), respectively). The participants indicated which treatments they would adopt to manage them. Participants were classified as ‘following’, ‘partially following’, ‘partially not following’ and ‘not following’ the CPGs and recommendations. In the third section, participants expressed their agreement with different CPG and recommendation statements through a 1-5 Likert scale.

**Results:**

In total, 483 physiotherapists (age: 34 ± 10; female 38%, male 61.5%, other 0.5%) answered the survey: 85% completed the first two sections, 76% completed all three sections. In a case of acute LAS with negative OAR, 4% of the participants were considered as ‘following’ recommended treatments, 68% as ‘partially following’, 23% as ‘partially not following’, and 5% as ‘not following’. In a case of acute LAS with positive OAR, 37% were considered ‘following’ recommended treatments, 35% as ‘partially following’, and 28% as ‘not following’. In the third section, the consensus was achieved for 73% of the statements.

**Conclusion:**

This study showed that although there is a good knowledge about first-line recommended treatments, a better use of CPGs and recommendations should be fostered among physiotherapists. Our results identify an evidence-to-practice gap in LAS management, which may lead to non-evidence-based practice behaviors.

**Supplementary Information:**

The online version contains supplementary material available at 10.1186/s12891-022-05914-5.

## Introduction

Lateral ankle sprains (LASs) are the most common lower limb musculoskeletal (MSK) injuries in sports and recreational physical activities [1]. LASs have a high prevalence both in the active and in the general population, leading to substantial healthcare burden [2]. Nonetheless, LASs are often considered innocuous injuries that will heal expediently and with minimal treatment [2, 3]. Physiotherapists are one of the main healthcare professionals who should take care of people with MSK diseases, including LASs, by providing evidence-based practice (EBP) treatments [4]. To facilitate the use of EBP in LAS management, several CPGs and Consensus Statements have been published in the last few years [4–7].

Once taking in charge a person with LAS, clinicians should start analysing LAS risk factors (e.g., an history of a previous LAS, type and level of sport practised, workload and level of participation, deficiencies in proprioception and ROM) as well as the risk factors for developing instability (e.g., the absence of balance or proprioception exercises following an acute lateral ankle sprain) [5, 6, 8]. Clinicians may incorporate in their assessment outcome measures for identifying the presence and severity of ankle instability (e.g., Cumberland Ankle Instability Tool [7]) and functionality (e.g., the Foot and Ankle Ability Measure [9] and the Lower Extremity Functional Scale [10]) [5, 6]. The ligament assessment is optimised once delayed for 4 to 5 days from the injury [8]. Finally, the Ottawa ankle rules (OAR) [11] should be used to determine whether a radiograph is required to rule out a fracture of the ankle and/or foot [4–6].

For what concerns the treatment, in the acute and protected motion phase, clinicians should advise the use of external supports for 4-6 weeks [8]. The device choice should be based on factors like the severity of the injury [5, 6]. People with LAS should bear more and more weight progressively on the affected limb through exercise therapy, no matter the severity [6]. Moreover, manual therapy techniques are recommended in adjunct to exercise (e.g., lymphatic drainage, active and passive soft tissue and joint mobilisation, and anterior-to-posterior talar mobilisation procedures) [4]. The use of repeated intermittent applications of ice is recommended to reduce pain [6]. It is not recommended to suggest rest, compression, and elevation alone [8]. Non-steroidal anti-inflammatory drugs may be used only to reduce pain and swelling [8]. There is weak and conflicting evidence on the use of diathermy, electrotherapy, and low-laser therapy, but it is certain that ultrasounds should not be adopted [5, 6].

However, the use of non-evidence treatments appears to be increasing among physiotherapists [12]. Moreover, knowledge of a recommendation and its application in clinical practice are not always consistent. This lack of this consistency is referred to as the ‘evidence-to-practice gap’. Different studies have addressed the knowledge of and adherence to CPGs for many MSK disorders, in different countries [13–18]. When it comes to LAS management, a few studies investigated either the knowledge of [18] or the adherence to LAS CPGs and recommendations in isolation [19, 20], with no studies focussed on the evidence-to-practice gap. Hence, this study investigated the ‘evidence-to-practice’ gap in CPGs and recommendations for LAS among Italian physiotherapists through a cross-sectional study design.

## Methods

### Study design

The present cross-sectional study is based on an online survey investigating Italian physiotherapists’ knowledge of and adherence to LAS CPGs and recommendations. The questionnaire was developed in Italian according to the *International Handbook of Survey Methodology* [21]*.* The study was conducted following the Declaration of Helsinki and ethical approval was obtained from the Research Ethics Committee of the University of Genoa (CERA: Comitato Etico per la Ricerca di Ateneo, approval date: 05/04/2021; n. 2021.40). This work is reported following the *Strengthening the Reporting of Observational Studies in Epidemiology* (STROBE) recommendations for reporting observational studies [22].

### Survey development

The questionnaire was divided into three sections: *section I – participants’ demographic characteristics (question 2 to question 11)*; *section II – clinical vignette (question 12 to question 13 - adherence*); *section III – statements consensus (question 14 to question 15 - knowledge*). Each section is thoroughly discussed below. The survey is available in English in the Supplementary file A (title: ‘Translated survey in English language’) and in Italian in the Supplementary file B (title: ‘Survey in Italian language’).

Both *section II* and *III* are based on the ‘Ankle Stability and Movement Coordination Impairments: Ankle Ligament Sprains Clinical Practice Guidelines Linked to the International Classification of Functioning, Disability and Health From the Orthopaedic Section of the American Physical Therapy Association’ [6] and on the ‘Diagnosis, treatment and prevention of ankle sprains: update of an evidence-based clinical guideline’ [8]. The most recent CPGs from the Orthopaedic Section of the American Physical Therapy Association were published in 2021 after the beginning of the study [23]. Therefore, we have analysed our data also in the light of this CPG, though the questionnaire was not created based on it.

The data for the investigation were collected through an electronic survey created with Microsoft 365 Forms, a secure web application to build and manage online surveys and databases, respecting the European General Data Protection [24]. Data were collected from May 2021 to August 2021. Before answering the survey, the participants were provided with an informed consent. Those who refused to give their consent to participate to the study were shown a “Thank-You page” and were not allowed to continue.

In the first section demographic data were collected (Table 3).

In the second section of the survey, the participants were asked to state how they would treat two clinical cases reported in two clinical vignettes (Table 1). Clinical vignettes are valid and acceptable tools to measure clinical decision making and observance of EBP guidelines [25]. Both vignettes represented a scenario in an acute and protected motion phase after an ankle injury. The first and the second vignettes reported negative and positive OAR for suspecting a bone fracture, respectively. The participants were asked to carefully read the vignettes and to select the therapeutic strategies they would apply in the first week of physiotherapy by selecting from a list of nineteen options that revolved around the assessment and management of the two simulated patients. The Supplementary file C (title: ‘evidence-based practice recommendations’) reports which were the recommended options from the CPGs and recommendations [6, 8].

**Table 1:** Section II: clinical vignette

***Vignette 1***: first episode of acute LAS with negative signs and symptoms for suspecting a bone fracture (negative OAR).	
History: A.R. is a 40-year-old woman, working as a post office employee with a passion for gardening. Yesterday she had a first episode of LAS when she put her foot in plantar flexion and inversion while gardening. She managed to go back home limping. The day after the injury, she went to the physiotherapist, walking with the help of two crutches and keeping her foot off the ground. Physical examination: When asked to put her foot on the ground to try to walk four steps, the patient stated that she was afraid of feeling pain, however she was able to walk throughout the room without limping, but with a pain in the lateral compartment of 4 out of 10 on the VAS (Visual Analogue Scale) pain scale. She has no pain on palpation of the posterior 6 cm of the malleoli, nor the lateral and medial midfoot area. There is mild oedema and haematoma in the anterolateral compartment of the ankle.	
***Vignette 2***: reinjury acute lateral ankle sprain with positive signs and symptoms for suspecting a bone fracture (positive OAR).	
History: G.C. is a 20-year-old female basketball player studying at university. Two days ago, during a game, she had an episode of LAS while placing her foot in plantar flexion and inversion when landing from a jump. This is the second episode of a sprained ankle injury, the first occurred three years ago, after which she underwent rehabilitation until she could play again. This time she had to stop playing immediately during the competition, came out from the basketball field hopping on the opposite foot. She applied ice immediately and the ankle got quickly swollen. She tried to put her foot on the floor and bare weight to walk to the changing room, but the pain was too high (VAS 8/10). Until now she has kept her foot elevated with ice and she never put it down on the floor to walk, but at night her ankle hurts a lot (VAS 8/10). She presented two days after the injury to the physiotherapist for the first visit, walking with two crutches without weight bearing. Physical examination: when asked to place her foot on the floor to try to walk 4 steps the patient reported 8 out of 10 pain on the VAS (Visual Analogue Scale) pain scale, by palpating the 6 cm posterior to the peroneal malleolus she reported a pain level of 7/10 on the VAS scale.	

In the third section the participants were asked to choose their level of agreement through a 1 (completely disagree) to 5-point (completely agree) Likert scale [26] to a total of eleven statements (Table 2).

**Table 2:** Section III: consensus statements

**Statements about assessment**	
1) The clinical assessment of damage to the ligaments after an ankle sprain should be performed within 24 hours from the trauma. (**Reversed statement**)	
2) In case of suspected fracture of the ankle or the foot, it is not recommended to apply the Ottawa ankle rules. (**Reversed statement**)	
3) During the anamnesis it is important to assess previous events of ankle sprains.	
4) In front of a second episode of lateral ankle sprain it is never necessary to apply the Ottawa ankle rules. (**Reversed statement**)	
5) Physiotherapists should incorporate functional outcome measures such as the FAAM (Foot and Ankle Ability Measure), as part of the examination of people with ankle sprain.	
**Statements about treatment**	
6) In front of recurrent ankle sprains, the clinician should recommend to follow a therapeutic exercise programme for coordination and balance for at least 1 year from the trauma.	
7) The brace has a role in the prevention of recurrent lateral ankle sprains events.	
8) At list one of the following treatment modalities is strongly recommended for the management of patients with ankle sprain during the acute phase: ultrasound, laser therapy, electrotherapy, diathermy. (**Reversed statement**)	
9) In the treatment of people with an ankle sprain, clinicians should use manual therapy procedures, such as lymphatic drainage, joint and soft tissue mobilisation.	
10) For people with severe ankle sprains, physiotherapists should implement rehabilitation programmes that include therapeutic exercises.	
11) When evaluating the results of the rehabilitation programme for an ankle sprain, physiotherapists should plan a follow-up until one year since the trauma.	

Participants were considered to agree with the statements if their score was 4-5; conversely, they were considered to disagree with the statements if their score was 1-3. Furthermore, to limit acquiescence bias, i.e., the tendency to agree with all the survey statements [27], four reversed statements were put into the questionnaire so that disagreement with those statements (scores 1–2) would indicate an agreement with the CPGs and recommendations [16, 28]. Each statement was acquired from the review of the CPGs [5, 6] and Consensus Statement [8] and the expected answers are represented in the Supplementary file C.

### Participants

The participants were recruited through different ways. Firstly, by receiving an e-mail with the hyperlink to the questionnaire through the Italian Association of Italian Physiotherapists (AIFI: *Associazione Italiana di Fisioterapia*) and newsletter of the 1st level Master in MSK disorders rehabilitation of the University of Genova. Secondly, they were contacted directly by the authors or through social media outlets.

To be included in the study the participants had to give their consent to partake in the study. To be eligible, the participants had to: (1) own a BSc in Physiotherapy obtained in Italy and be currently working as a physiotherapist in Italy; (2) have seen at least one person with an ankle sprain during the previous 2 years. Those who gave a negative answer at these two questions, were sent at the end of the survey, and could not proceed with the questions.

### Variables

The primary outcome of the current investigation was to describe the knowledge of and adherence to the CPGs and recommendations for LASs in a sample of Italian physiotherapists. This allowed the authors to identify any possible ‘evidence-to-practice gap’ [29].

### Statistical Analysis

The data about the demographic section collected through multiple choice questions were reported as presented in an Excel file. The demographics (age, gender, years of practice, highest academical title achieved) were analysed through descriptive analysis calculating mean, frequencies, and standard deviations. Continuous variables were reported as mean ± standard deviation (SD), while categorical variables were reported as absolute and percentage frequencies.

In the statistical analysis of the *section II* about adherence investigation the participants were divided into four subcategories: (1) ‘following’, (2) ‘partially following’, (3) ‘partially not following’ and (4) ‘not following’ the CPGs and recommendations based on their answers as described hereafter. The authors’ comments and choices have been specified in detail in the Supplementary file C.

Regarding the *vignette 1*, where the information of the text clearly says that the patient was presenting negative OAR, the participants were considered as: (1) ‘following’ the recommendations if they chose *only* treatments with a high level of recommendations (Grade A or Level 1); (2) ‘partially following’ the recommendations if they chose treatments that have a high level of recommendations (Grade A or Level 1), together with treatments that have a lower level of recommendations (Grade B-C or Level 2); (3) ‘partially not following’ the recommendations if they chose *only* treatments that have a low grade of recommendation (Grade C-D-E-F or Level 2-3-4); (4) ‘not following’ the recommendations if their choices included treatments that are not recommended to be used (e.g. ultrasound therapy, Grade A), whether alone or in combination with other treatments.

As mentioned above, *vignette 2* showed a person presenting positive OAR. The participants were considered according to their adherence at the use of the OAR as: (1) ‘following’ when they only chose the option to ‘contact the specialist or to go to the emergency room’; (2) ‘partially following’ if they chose to apply components of RICE (Rest, Ice, Compression and Elevation), referral to the doctor for FANS and the use of a brace but only in addition to ‘contact the specialist or to go to the emergency room’; (3) ‘not following’ the correct use of the OAR every time they did not include in their choice to refer the patient to the specialist or to the emergency room or they did the referral but they also started a treatment, without excluding possible bone fractures before. Considering the specific emergency scenario, the group ‘partially not following’ was not created.

*Section III* was analysed to understand the level of agreements of the participants to the statements retrieved from CPGs and recommendations. In the not reversed statements, answering 1 (‘completely agree’) and 2 (‘partially agree’) on the 5-point Likert scale were considered in agreement with the statements. Conversely, answering 3 (‘neither agree nor disagree’), 4 (‘partially disagree’) and 5 (‘completely disagree’) on the 5-point Likert scale was considered in disagreement with the EBP recommendations. To what concerns the reversed statements, answering 4 (‘partially disagree’) and 5 (‘completely disagree’) on the 5-point Likert scale was considered in agreement with the EBP recommendations. Hence, answering 1 (‘completely agree’), 2 (‘partially agree’) and 3 (‘neither agree nor disagree’) on the 5-point Likert scale were considered in disagreement with the EBP recommendations. In the absence of a standard threshold, the authors defined a ≥ 70% agreement with a statement as consensus [13, 29]. The frequencies of answers were calculated and a visual representation through a bar chart graph is reported in Fig. 2.

### Study size

To determine the sample size for this online survey, the formula reported by Taherdoost et al. [30] was applied. Specifically, the sample size was the number of completed responses expected to be received. The calculated sample size necessary for this study was of 370 taking into consideration the number of Italian physiotherapists enrolled in the Italian professional register, following the formula, setting a 5% margin of error (how accurately the results of the survey would reflect the views of the general population) and a sampling confidence level of 95% (how confident the authors could be that the population would select an answer within a certain range).

## Results

### Participants

**Fig. 1:**
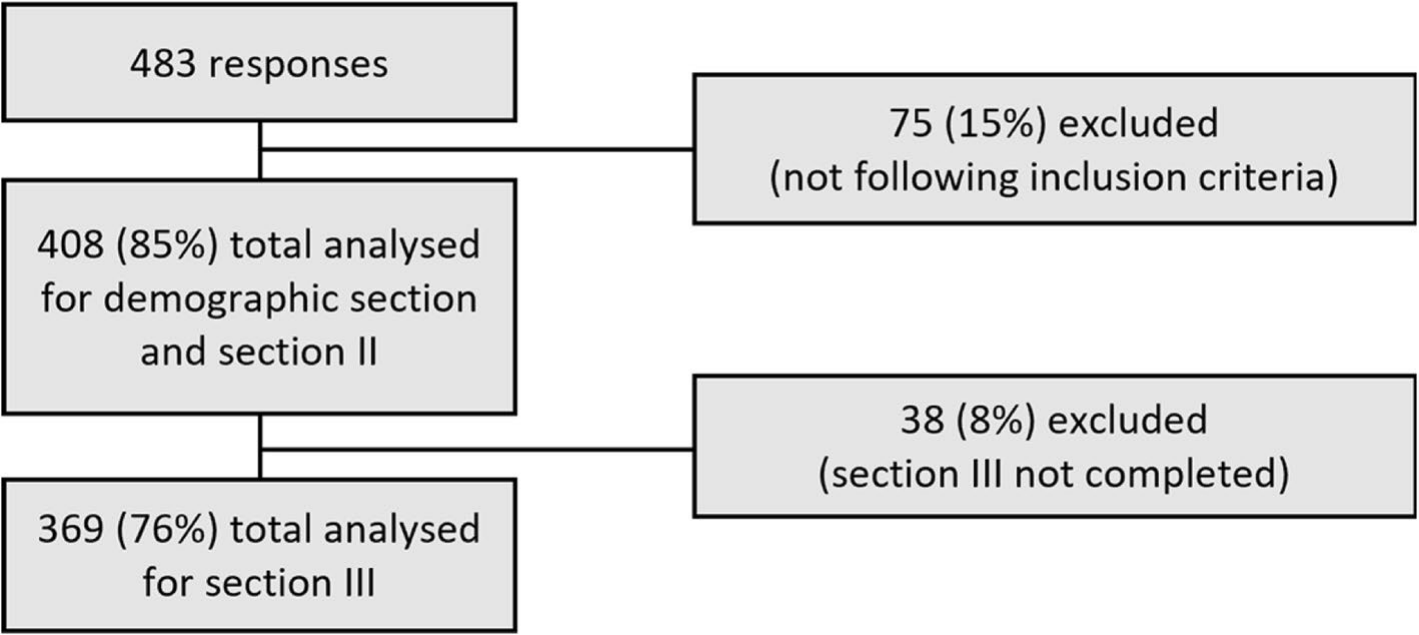
Participants’ flowchart

Through the AIFI and the University of Genova newsletter, and dissemination via social media outlets, the authors were able to collect a total of 483 responses in a period from May 2021 to August 2021. Among them, 11 (2%) did not accept to partake in the survey after reading the informed consent, 26 (5%) did not graduate in Italy or were not currently working as Physiotherapist in Italy, and 38 (8%) did not treat any person with a LAS in the previous 2 years. Those who completed the *section I* and the *section II* are 408 (85%; mean age (SD): 34 (10); female 38%, male 61.5%, other 0.5%), and those that had completed the survey in all its sections are 369 (76%) (Fig. 1).

The demographics of the participants are displayed in Table 3.

**Table 3:** Participants’ profile by level of adherence

		clinical ***vignette 1***				clinical ***vignette 2***		
Groups (N)	Total eligible participants (408)	“Following” (17)	“Partially Following” (278)	“Partially Not Following” (93)	“Not Following” (20)	“Following” (151)	“Partially Following” (143)	“Not Following” (114)
**Age** (years (mean (SD)))	34 (10)	32 (9.5)	32 (7.6)	39 (12.0)	41 (12.7)	33 (9.3)	33 (9)	37 (10.6)
**Gender** (N (%)):								
Female	155 (38)	5 (29)	95 (34)	45 (48)	10 (50)	49 (32)	58 (41)	48 (42)
Male	251 (61.5)	12 (71)	181 (65)	48 (52)	10 (50)	102 (68)	85 (59)	64 (56)
Other	2 (0.5)	0 (0)	2 (1)	0 (0)	0 (0)	0 (0)	0 (0)	2 (2)
**Years of practice** (N (%)):								
Less than 1 year	22 (5)	2 (12)	15 (5)	3 (3)	2 (11)	9 (6)	7 (5)	6 (5)
From 1 to 5 years	150 (37)	7 (41)	117 (42)	22 (24)	4 (21)	61 (40)	58 (41)	31 (27)
From 6 to 10 years	100 (25)	3 (18)	68 (25)	22 (24)	7 (37)	42 (28)	32 (22)	26 (23)
More than 10 years	136 (33)	5 (29)	78 (28)	46 (49)	7 (31)	39 (26)	46 (32)	51 (45)
**Highest academical education reached** (N (%)):								
Bachelor of Science (BSc)	223 (54.8)	12 (71)	154 (55)	59 (64)	18 (89)	85 (56)	83 (58)	75 (66)
Post-Graduate I Level Degree*	153 (37)	3 (18)	108 (39)	27 (29)	2 (11)	55 (36)	52 (36)	33 (29)
Master of Science (MSc) /Post-Graduate II Level Degree**	31 (8)	2 (12)	16 (6)	6 (6)	0 (0)	11 (8)	8 (6)	5 (4)
Doctor of Philosophy (PhD)	1 (0.2)	0 (0)	0 (0)	1 (1)	0 (0)	0 (0)	0 (0)	1 (1)
**Participation to a course on the topic ‘LAS’** (N (%)):								
Yes	129 (32)	5 (29)	86 (31)	34 (37)	5 (21)	48 (32)	45 (32)	36 (32)
No	279 (68)	12 (71)	192 (69)	59 (63)	15 (79)	103 (68)	98 (68)	78 (68)

*N* Number, *SD* Standard deviation; %, percentage; *Academic degree that can be gained after BSc (Italian education system); ** Academic degree that can be gained after MSc (Italian education system)

Participants’ demographic profiles divided in ‘following’, ‘partially following’, ‘partially not following’ and ‘not following’ are reported in Table 3 for the clinical *vignette 1* and for the clinical *vignette 2*. The percentages of the selection of each item in the groups is reported in Table 4 for the clinical *vignette 1* and in Table 5 for the clinical *vignette 2*.

**Table 4:** Frequencies of answers to clinical *vignette 1* by level of adherence to Clinical Practice Guidelines

Groups (N (%))	All (408 (100))		“Following” (17 (4))		“Partially Following” (278 (68))		“Partially Not Following” (93 (23))		“Not Following” (20 (5))	
	Yes	No	Yes	No	Yes	No	Yes	No	Yes	No
Application of ice/cryotherapy alone	37 (9)	371 (91)	0 (0)	17 (100)	12 (4)	266 (96)	19 (20)	74 (80)	6 (30)	14 (70)
Application of ice/cryotherapy in combination with tolerated active mobilisation	297 (73)	111 (27)	0 (0)	17 (100)	222 (80)	56 (20)	64 (69)	29 (31)	11 (55)	9 (45)
Compression	256 (63)	152 (37)	0 (0)	17 (100)	194 (70)	84 (30)	50 (54)	43 (46)	12 (60)	8 (40)
Elevation	265 (65)	143 (35)	0 (0)	17 (100)	202 (72)	76 (28)	52 (56)	41 (44)	11 (55)	9 (45)
Protection with a semi-rigid brace	67 (16)	341 (84)	2 (12)	15 (88)	61 (22)	217 (78)	0 (0)	93 (100)	4 (20)	16 (80)
Protection with a lace-up brace	43 (11)	365 (89)	0 (0)	17 (100)	41 (15)	237 (85)	0 (0)	93 (100)	2 (10)	18 (90)
Protection with elastic tape (kinesiotape)	97 (24)	311 (76)	1 (6)	16 (94)	52 (19)	226 (81)	37 (40)	56 (60)	7 (35)	13 (65)
Advice to the patient to contact the specialist or to go to the emergency room	16 (4)	392 (96)	0 (0)	17 (100)	4 (1)	274 (99)	12 (13)	81 (87)	0 (0)	20 (100)
Advice to the patient to contact the specialist or to go to the emergency room, starting in the meantime the rehabilitation program	28 (7)	380 (93)	0 (0)	17 (100)	11 (4)	267 (96)	10 (11)	83 (89)	7 (35)	13 (65)
Referral of the patient to the doctor for a possible pharmacological treatment	16 (4)	392 (96)	0 (0)	17 (100)	9 (3)	269 (97)	6 (6)	87 (94)	1 (5)	19 (95)
Recommend to rest and immobilization for 2 weeks	2 (0.5)	406 (99.5)	0 (0)	17 (100)	0 (0)	278 (100)	0 (0)	93 (100)	2 (10)	18 (90)
Recommend for laser therapy	21 (5)	387 (95)	0 (0)	17 (100)	10 (4)	268 (96)	6 (6)	87 (94)	5 (25)	15 (75)
Recommend for diathermy	30 (7)	378 (93)	0 (0)	17 (100)	18 (6)	260 (94)	6 (6)	87 (94)	6 (30)	14 (70)
Recommend for antalgic electrotherapy	3 (1)	405 (99)	0 (0)	17 (100)	0 (0)	278 (100)	0 (0)	93 (100)	3 (15)	17 (85)
Recommend for ultrasound therapy	14 (3)	394 (97)	0 (0)	17 (100)	0 (0)	278 (100)	0 (0)	93 (100)	14 (70)	6 (30)
Passive joint mobilization with manual therapy techniques alone	20 (5)	388 (95)	0 (0)	17 (100)	9 (3)	269 (97)	9 (10)	84 (90)	2 (10)	18 (90)
Passive joint mobilization with manual therapy techniques in combination with other active treatments	211 (52)	197 (48)	13 (76)	4 (24)	188 (68)	90 (32)	0 (0)	93 (100)	10 (50)	10 (50)
Active mobility exercises	174 (43)	234 (57)	10 (59)	7 (41)	158 (57)	120 (43)	0 (0)	93 (100)	6 (30)	14 (70)
Exercises such as step up, squat, jumps and aerobic exercises	27 (7)	381 (93)	1 (6)	16 (94)	26 (9)	252 (91)	0 (0)	93 (100)	0 (0)	20 (100)

*N* Number; %, percentage; the percentage in the columns “Yes” and “No” is calculated on the N of the reference group

**Table 5:** Frequencies of answers to clinical *vignette 2* by level of adherence to Clinical Practice Guidelines

Groups (N (%))	All (408 (100))		“Following” (151 (37))		“Partially Following” (143 (35))		“Not Following” (114 (28))	
	Yes	No	Yes	No	Yes	No	Yes	No
Application of ice/cryotherapy alone	132 (32)	276 (68)	0 (0)	151 (100)	92 (64)	51 (36)	40 (35)	74 (65)
Application of ice/cryotherapy in combination with tolerated active mobilisation	32 (8)	376 (92)	0 (0)	151 (100)	0 (0)	143 (100)	32 (28)	82 (72)
Compression	139 (34)	269 (66)	0 (0)	151 (100)	79 (55)	64 (45)	60 (53)	54 (47)
Elevation	171 (42)	237 (58)	0 (0)	151 (100)	97 (68)	46 (32)	74 (65)	40 (35)
Protection with a semi-rigid brace	123 (30)	285 (70)	0 (0)	151 (100)	71 (50)	72 (50)	52 (46)	62 (54)
Protection with a lace-up brace	17 (4)	391 (96)	0 (0)	151 (100)	9 (6)	134 (94)	8 (7)	106 (93)
Protection with elastic tape (kinesiotape)	18 (4)	390 (96)	0 (0)	151 (100)	0 (0)	143 (100)	18 (16)	96 (84)
Advice to the patient to contact the specialist or to go to the emergency room	328 (80)	80 (20)	151 (100)	0 (0)	143 (100)	0 (0)	34 (30)	80 (70)
Advice to the patient to contact the specialist or to go to the emergency room, starting in the meantime the rehabilitation program	67 (16)	341 (84)	0 (0)	151 (100)	0 (0)	143 (100)	67 (59)	47 (41)
Referral of the patient to the doctor for a possible pharmacological treatment	19 (5)	389 (95)	0 (0)	151 (100)	11 (8)	132 (92)	8 (7)	106 (93)
Recommend to rest and immobilization for 2 weeks	7 (2)	401 (98)	0 (0)	151 (100)	0 (0)	143 (100)	7 (6)	107 (94)
Recommend for laser therapy	11 (3)	397 (97)	0 (0)	151 (100)	0 (0)	143 (100)	11 (10)	103 (90)
Recommend for diathermy	14 (3)	394 (97)	0 (0)	151 (100)	0 (0)	143 (100)	14 (12)	100 (88)
Recommend for antalgic electrotherapy	6 (1)	402 (99)	0 (0)	151 (100)	0 (0)	143 (100)	6 (5)	108 (95)
Recommend for ultrasound therapy	6 (1)	402 (99)	0 (0)	151 (100)	0 (0)	143 (100)	6 (5)	108 (95)
Passive joint mobilization with manual therapy techniques alone	14 (3)	394 (97)	0 (0)	151 (100)	0 (0)	143 (100)	14 (12)	100 (88)
Passive joint mobilization with manual therapy techniques in combination with other active treatments	17 (4)	391 (96)	0 (0)	151 (100)	0 (0)	143 (100)	17 (15)	97 (85)
Active mobility exercises	11 (3)	397 (97)	0 (0)	151 (100)	0 (0)	143 (100)	11 (10)	103 (90)
Exercises such as step up, squat, jumps and aerobic exercises	5 (1)	403 (99)	0 (0)	151 (100)	0 (0)	143 (100)	5 (4)	109 (96)

*N* Number; %, percentage; the percentage in the columns “Yes” and “No” is calculated on the N of the reference group

Regarding the *vignette 1*, where a person with negative OAR is presented, 17 participants (4%) were in the ‘following’ group and provided only high level recommended treatments (i.e., protection with a semi-rigid brace and exercise, passive joint mobilisation with manual therapy techniques in combination with other active treatments and active mobility exercises). Then, 278 physiotherapists (68%) were considered as ‘partially following’ since they chose high level recommended choices such as mobility exercises (Grade A) in combination with low level recommendations, as laser therapy (Grade C-D). Most of the sample in this group (80%) applied ice/cryotherapy in combination with tolerated active mobilisation. The group ‘partially not following’ is made of 93 physiotherapists (23%) who provided the patient only with low level recommendations, such as to use diathermy (Level 2, Grade C) and components of RICE (Level 2). In the ‘not following’ group 20 physiotherapists (5%) chose treatments that are recommended to avoid, such as ultrasound therapy (Grade A for not to be used) (74% of the sample), resting and immobilisation for 2 weeks (6%), or only not recommended treatments, such as components of RICE alone.

As far as the *vignette 2* is concerned, where a patient with positive OAR was presented, 151 physiotherapists (37%) ended up in the ‘following’ group, referring the patient to a specialist or to the emergency. The ‘partially following group’ is made of 145 physiotherapists (35%) who correctly chose to refer the person to the specialist or to the emergency room (100%), but they added other treatments who are not the first choice for these types of patients such as recommending elevation (Level 2) (68%) or application of ice (Grade C, Level 2) (64%). Finally, the 112 participants (28%) were considered as ‘not following’ since they did not consider necessary to refer the patients to a specialist or to the emergency room, or they would start a rehabilitation programme without ruling out the presence of fractures first.

**Fig. 2:**
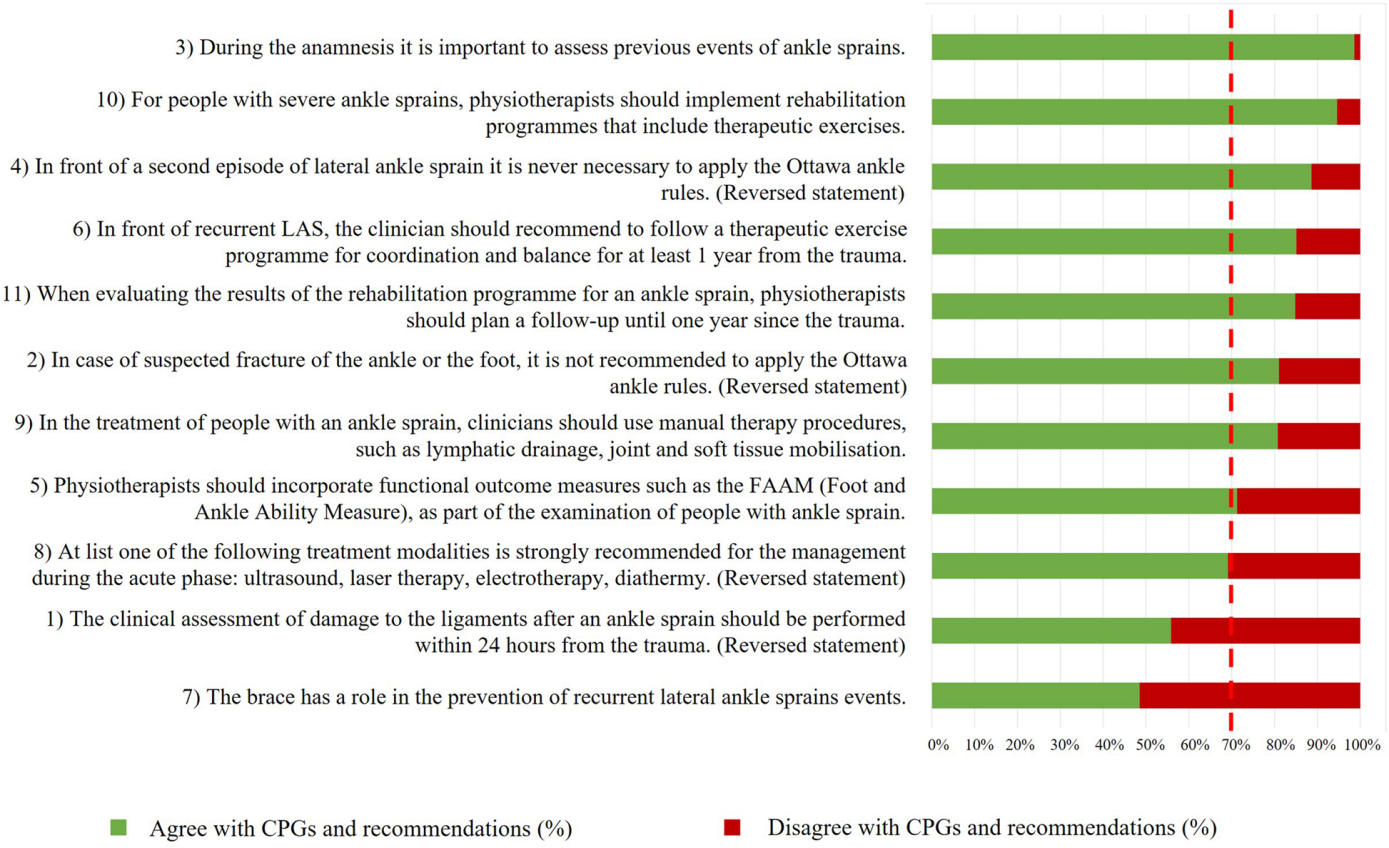
Reported answers to *section III* and consensus at 70%. Due to graphical reasons some statements have been shortened; the complete sentences are available in Table 2

Considering that 369 participants completed the *section III*, the percentages of agreement and disagreement in the answers on a 5-point Likert scale are reported in the Table 6. The Fig. 2 represent for which statements the consensus was reached at 70%.

**Table 6:** *Section III*: level of agreement on a 5-point Likert scale (tot *N* = 369)

Statements about assessment	Agreement (N (%))	Disagreement (N (%))
1) The clinical assessment of damage to the ligaments after an ankle sprain should be performed within 24 hours from the trauma. (**Reversed statement**)	206 (56)	163 (44)
2) In case of suspected fracture of the ankle or the foot, it’s not recommended to apply the Ottawa ankle rules. (**Reversed statement**)	299 (81)^a^	70 (19)
3) During the anamnesis it is important to assess previous events of ankle sprains.	364 (99)^a^	5 (1)
4) In front of a second episode of lateral ankle sprain it is never necessary to apply the Ottawa ankle rules. (**Reversed statement**)	327 (89)^a^	42 (11)
5) Physiotherapists should incorporate functional outcome measures such as the FAAM (Foot and Ankle Ability Measure), as part of the examination of patients with ankle sprain.	163 (71)^a^	106 (29)
**Statements about treatment**	**Agreement** (N (%))	**Disagreement** (N (%))
6) In front of recurrent ankle sprains, the clinician should recommend to the patent to follow a therapeutic exercise program for coordination and balance for at least 1 year from the trauma.	314 (85)^a^	55 (15)
7) The brace has a role in the prevention of recurrent lateral ankle sprains events.	179 (49)	190 (51)
8) At list one of the following treatment modalities is strongly recommended for the management of patients with ankle sprain during the acute phase: ultrasound, laser therapy, electrotherapy, diathermy. (**Reversed statement**)	225 (69)	114 (31)
9) In the treatment of patients with an ankle sprain, clinicians should use manual therapy procedures, such as lymphatic drainage, joint and soft tissue mobilization.	298 (81)^a^	71 (19)
10) For patients with severe ankle sprains, physiotherapists should implement rehabilitation programs that include therapeutic exercises.	349 (95)^a^	20 (5)
11) When evaluating the results of the rehabilitation program for an ankle sprain, physiotherapists should plan a follow-up until one year since the trauma.	313 (85)^a^	56 (15)

*N* Number; %, percentage for the statements the answers of agreement are 4 or 5 on a 5-point Likert scale; reversed statement = the answers of agreement are 1 or 2 on a 5-point Likert scale; ^a^ the consensus is > 70%

Overall, consensus to the statements was achieved for 8 (73%) statements (2, 3, 4, 5, 6, 9, 10, 11) out of 11. Among the statements that found a consensus among the participants, 4 investigated the assessment phase, including the procedures to rule in or out a bone fracture, risk factors such as previous ankle injuries, the use of patient’s reported outcomes; 4 investigated the treatments choices, including the use of manual therapy, the use of therapeutic exercise and the duration of the therapy program until the last follow-ups. Conversely, the consensus was not achieved for 3 (27%) statements (1, 7, 8). These statements revolve around the time within the clinical assessment should be performed, the possible function of prevention of brace, the level of recommendation for physical therapies such as laser therapy and ultrasound.

## Discussion

CPGs and evidence-based recommendations are essential to convey EBM treatments. Physiotherapists should know and translate these recommendations in their clinical practice. However, knowledge of a recommendation and its application in the clinical practice are not always consistent and this is referred to as the ‘evidence-to-practice gap’ [12, 31]. When it comes to LAS management, Italian physiotherapists seem to be aware of the highly recommended treatments in case of an acute LAS injury and they seemed to apply them in their clinical practice. An evidence-based assessment and rehabilitation is essential for people with LAS to return to previous levels of work, sport, and normal physical activities. The effect of this narrow evidence-to-practice bridge can optimise the recovery of joint functionality in people with LAS and lower the risk of recurrent ankle sprains, lowering the risk of developing chronic ankle instability (CAI) [6, 8, 23, 28, 32].

In our sample, highly recommended treatments sometimes were applied together with therapies with a lower level of recommendation such as the use of physical therapies, as diathermy (7%), laser-therapy (5%), electrotherapy (1%), and ultrasounds (3%). However, when we asked them whether they considered these physical therapies as non-recommended treatments, we did not reach a consent (69%), showing a gap between their levels of adherence and knowledge. Moreover, highly recommended treatments were often applied together with some components of RICE, which is a lower recommended choice. Finally, still a 23% of the participants would have applied only a combination of treatments that should not be the first line choice after a LAS.

The reason behind keep using some lower recommended treatments in the clinical practice might stem from an attempt to meet patients’ expectations and values, which are also part of the EBP principles [4]. However, providing only low recommended treatments in people with LAS, such as the use of RICE alone, or the use of not recommended therapies such as ultrasounds therapy can lead to the development of long-term symptoms, causing a decrease in physical activity and quality of life [2, 28, 32, 33]. Moreover, this bad practice can also increase the economic burden related to the direct costs of long term treatments and the indirect costs of work-related time loss [2]. Therefore, physiotherapists should investigate patients’ expectations related to the treatments and providing education strategies to reduce the gap between what patients want and actually need [34].

When it comes to emergency context, Italian physiotherapists seem to know they should apply OAR when a bone fracture is suspected after an episode of LAS, and they also apply these rules in clinical practice. In the clinical vignette, two third of the physiotherapists correctly recognised the positivity of the OAR, referring the patient to a physician. However, there are still one third of the sample that did not recognise them in a clinical scenario. This study shows also that the participants do not know when a ligament evaluation should be done after LAS, even if the literature recommend to do it between four and 5 days after injury [8]. Physiotherapists working with MSK disorders should own advanced assessment skills to conduct a complex evaluation and refer the patients to other professional figures when necessary. This is even more fundamental when it comes to direct access, where the physiotherapist is the first healthcare professional that the patient refers to [35]. More and more evidence is showing that direct access services to physiotherapy in MSK disorders care might help to manage physicians’ workload whilst leading to similar clinical outcomes, reducing resources that could be saved and spent in other healthcare areas [35–37]. Therefore, it is important for the physiotherapists to develop advanced reasoning skills level and clinical knowledge needed to understand when the conditions they are assessing do not pertain to them.

Between the barriers that limit the application of EBP in the management of people with LAS could be the fact that it is often considered as an innocuous injury that will heal expediently and with minimal treatment [2, 3]. Therefore, physiotherapists might underestimate the impact of this injury which have important consequences of people’s life. Other barriers to EBP in physiotherapy could be the lack of time, problems in understanding statistical data, lack of support in the EBM implementation from employer(s) and colleagues [38]. In two studies by Castellini et al. and Cutolo et al., Italian physiotherapists overrated their level of knowledge of EBP and Scientific English, reporting inadequate levels thereof [39, 40]. As highlighted by Battista et al., some strategies to bridge the above-mentioned gap could be to improve the knowledge of Scientific English, the creation of CPGs in Italian language and their use in university programmes [13]. Nonetheless, post-professional specialisation and continuing medical education remain a pillar for developing advanced clinical, decision-making, and reasoning skills level [35].

Some limitations of this study need to be discussed. Firstly, this is an observational study with descriptive statistics. Future studies with different and more complex design (i.e., qualitative and mixed-method studies) should investigate the reasons behind the failure of the implementation of LAS CPGs and recommendations both from Italian physiotherapists’ and patients’ perspectives. Secondly, we did not investigate the participants’ clinical practice setting (i.e., private practice, public care etc.) and main area of specialisation (MSK, neurologic etc.) which might have had an impact on their level of knowledge of LAS CPGs and recommendations. It has also to be taken into consideration that the results of this study could represent a specific population between Italian physiotherapists since it is most likely that the majority of the participants received the questionnaire through the newsletter of the Italian Association of Italian Physiotherapists and of the 1st level Master in MSK disorders rehabilitation of the University of Genova, therefore they could be specialists in MSK rehabilitation or being actual students for this specialisation. Furthermore, we did not ask how much importance they gave to each treatment. Therefore, we could not understand whether they would prioritise one treatment among the others and the time they would allocate to it. Finally, it was not possible to calculate the response rate as this questionnaire was delivered through social media outlets. However, the necessary sample size was reached.

## Conclusion

The findings of this study highlight that the Italian physiotherapists are aware of first-line recommended treatments for acute LAS management. They seem to know that manual therapy mobilisation along with active exercises are the best practice in acute LAS management. However, they would still provide some not-recommended or partially recommended treatments. Moreover, one-third of our sample was not able to recognise a positive OAR. A better knowledge and use of CPGs in the physiotherapists’ clinical practice need to be fostered. By doing so, we will improve the quality of care delivered by physiotherapists, enhancing the quality of life and the levels of activity of our patients.

## Supplementary Information

**Additional file 1.**

**Additional file 2.**

**Additional file 3.**

## Data Availability

The datasets used and analyzed during the current study are available from the corresponding author on reasonable request.
