# Supplementary material for: Physiotherapists’ knowledge of and adherence to evidence-based practice guidelines and recommendations for ankle sprains management: a cross-sectional study
Source: BMC Musculoskelet Disord. 2022 Nov 11;23:975. doi: 10.1186/s12891-022-05914-5 (PMC9650827; doi:10.1186/s12891-022-05914-5)
Supplement: Supplementary file 1 — Additional file 1. [file 12891_2022_5914_MOESM1_ESM.docx]

**Supplementary File A**

**Translated survey in English language**

Physiotherapists’ Knowledge of and Adherence to Evidence-Based Practice Guidelines and Recommendations for Ankle Sprains Management: a Cross-Sectional Study

***Section I: participants’ demographic characteristics***

Dear Participant, you have been asked to take part in a study entitled "Physiotherapists’ Knowledge of and Adherence to Evidence-Based Practice Guidelines and Recommendations for Ankle Sprains Management: a Cross-Sectional Study".

Before you answer this survey, it is important that you understand the reason for the study and what you will be asked to do. The investigators are at your disposal for any clarification. The first two sections of this questionnaire are intended to provide you with correct and complete information so that you can make a free and informed choice. The person in charge of the study is Marco Testa, Aggregate Professor at the Department of Neuroscience, Rehabilitation, Ophthalmology, Genetics and Maternal and Child Sciences at the University of Genoa.

We thank you for your valuable contribution, if you have any questions please contact us by e-mail at giuliacaffini95@gmail.com

**A. Information about the research and informed consent**

1. The participant declare to have read and understood the "Study information file" at the link bit.ly/2QEBCbv : YES / NO

2. The participant declare to have read and understood the "Information on the processing of personal data” (ex artt. 9 e 10 del Reg. UE n. 2016/679)" at the link bit.ly/2RXmSEX and declare to give his/her consent to the University of Genoa to use his/her personal data for the purposes and with the modalities described in the document: YES / NO

**B. Participants’ demographic characteristics**

The participant is kindly requested to answer the following questions:

3. Have you graduated in Physiotherapy in Italy and are you currently working as a Physiotherapist in Italy? YES / NO

4. Have you treated at least a patient with an ankle sprain in the last two years? YES / NO

5. Age in number (e.g. 38): _____

6. Gender of identification: male / female / other*

*7. Specify the identification gender: ______

8. Years of work since graduation:

- Less than 1 year
- 1 to 5 years
- 6 to 10 years
- More than 10 years

9. Choose the academical education pathway title that you have obtained so far (more choices available):

- Bachelor of Science (BSc)
- Post-Graduate I Level Degree
- Master of Science (MSc) / Post-Graduate II Level Degree
- Doctor of Philosophy (PhD)
- Other to be specify*

*10. Specify next if you obtained more than one level of graduation (e.g. "Post-Graduate I Level Degree in Sport Physiotherapy"): ___________________________________________________________

11. Have you ever attended any specific course or seminary on the topic “rehabilitation of patients with ankle sprain”? YES / NO

***Section II: clinical vignette – adherence investigation***

Di seguito trova due casi clinici. La preghiamo di leggerli attentamente e di scegliere quali sono le procedure che metterebbe in atto per la gestione del paziente in prima settimana.

12. Vignette 1

Clinical scenario 1: first episode of acute lateral ankle sprain with negative signs and symptoms for suspecting a bone fracture, acute phase.

History: A.R. is a 40-year-old woman, working as a post office employee with a passion for gardening. Yesterday she suffered a first episode of lateral ankle sprain when she put her foot in plantar flexion and inversion while in the garden. She managed to limp home. The day after the injury she went to the physiotherapist, walking with the help of two crutches and keeping her foot off the ground.

Physical examination: When asked to put her foot on the ground to try to walk 4 steps, the patient stated that she was afraid of feeling pain, however she was able to walk throughout the room without limping, but with a pain in the lateral compartment of 4 out of 10 on the VAS (Visual Analogue Scale) pain scale. She has no pain on palpation of the posterior 6 cm of the malleoli, nor the lateral and medial midfoot area. There is mild oedema and haematoma in the anterolateral compartment of the ankle.

Please choose from the following list which procedures you would implement to manage the patient in this scenario in week 1 (more options available)

- Application of ice/cryotherapy alone
- Application of ice/cryotherapy in combination with tolerated active mobilization
- Compression
- Elevation
- Protection with a semi-rigid brace
- Protection with a lace-up brace
- Protection with elastic tape (kinesiotape)
- Advice to the patient to contact the specialist or to go to the emergency room
- Advice to the patient to contact the specialist or to go to the emergency room, starting in the meantime the rehabilitation program
- Referral of the patient to the doctor for a possible pharmacological treatment
- Recommend to rest and immobilization for 2 weeks
- Recommend for laser therapy
- Recommend for diathermy
- Recommend for antalgic electrotherapy
- Recommend for ultrasound therapy
- Passive joint mobilization with manual therapy techniques alone
- Passive joint mobilization with manual therapy techniques in combination with other active treatments
- Active mobility exercises
- Exercises such as step up, squat, jumps and aerobic endurance

13. Vignette 2

Clinical scenario 2: reinjury acute phase lateral ankle sprain with positive signs and symptoms for suspecting a bone fracture.

History: G.C. is a 20-year-old female basketball player studying at university. Two days ago, during a game, she suffered an episode of ankle sprain while placing her foot in plantar flexion and inversion when landing from a jump. This is the second episode of a sprained ankle injury, the first having occurred three years ago, after which she underwent rehabilitation until she could play again.

This time she had to stop the game, came out hopping on the opposite foot, applied ice immediately and the ankle got quickly swollen. She tried to put her foot on the floor and bare weight to walk to the changing room, but the pain was too high (VAS 8/10).

Until now she has kept her foot elevated with ice and she has not put it down on the floor to walk, but at night her ankle hurts (VAS 8/10). She presented two days after the injury to the physiotherapist for the first visit, walking with two crutches without weight bearing.

Physical examination: when asked to place her foot on the floor to try to walk 4 steps the patient reported 8 out of 10 pain on the VAS (Visual Analogue Scale) pain scale, by palpating the 6 cm posterior to the peroneal malleolus she reported a pain level of 7/10 VAS.

Please choose from the following list which procedures you would implement to manage the patient in this scenario in week 1 (more options available)

- Application of ice/cryotherapy alone
- Application of ice/cryotherapy in combination with tolerated active mobilization
- Compression
- Elevation
- Protection with a semi-rigid brace
- Protection with a lace-up brace
- Protection with elastic tape (kinesiotape)
- Advice to the patient to contact the specialist or to go to the emergency room
- Advice to the patient to contact the specialist or to go to the emergency room, starting in the meantime the rehabilitation program
- Referral of the patient to the doctor for a possible pharmacological treatment
- Recommend to rest and immobilization for 2 weeks
- Recommend for laser therapy
- Recommend for diathermy
- Recommend for antalgic electrotherapy
- Recommend for ultrasound therapy
- Passive joint mobilization with manual therapy techniques alone
- Passive joint mobilization with manual therapy techniques in combination with other active treatments
- Active mobility exercises
- Exercises such as step up, squat, jumps and aerobic endurance

***Section III: statements consensus – knowledge investigation***

Please read the next statements carefully and choose how much you agree with them, choosing a number from 1 (completely disagree) to 5 (completely agree).

14. How much do you agree with the following statements about assessment?

|  | 1  Completely disagree | 2  Partially disagree | 3  Neither agree nor disagree | 4  Partially agree | 5  Completely agree |
| --- | --- | --- | --- | --- | --- |
| The clinical assessment of damage to the ligaments after an ankle sprain should be performed within 24 hours from the trauma. |  |  |  |  |  |
| In case of suspected fracture of the ankle or the foot, it’s not recommended to apply the Ottawa ankle rules. |  |  |  |  |  |
| During the anamnesis it is important to assess previous events of ankle sprains. |  |  |  |  |  |
| In front of a second episode of lateral ankle sprain it is never necessary to apply the Ottawa ankle rules. |  |  |  |  |  |
| Physiotherapists should incorporate functional outcome measures such as the FAAM (Foot and Ankle Ability Measure), as part of the examination of patients with ankle sprain. |  |  |  |  |  |

15. How much do you agree with the following statements about treatment?

|  | 1  Completely disagree | 2  Partially disagree | 3  Neither agree nor disagree | 4  Partially agree | 5  Completely agree |
| --- | --- | --- | --- | --- | --- |
| When evaluating the results of the rehabilitation programme for an ankle sprain, physiotherapists should plan a follow-up until one year since the trauma |  |  |  |  |  |
| Both tape and brace have a role in the prevention of recurrent lateral ankle sprains events. |  |  |  |  |  |
| At list one of the following treatment modalities is strongly recommended for the management of patients with ankle sprain during the acute phase: ultrasound, laser therapy, electrotherapy, diathermy. |  |  |  |  |  |
| In the treatment of patients with an ankle sprain, clinicians should use manual therapy procedures, such as lymphatic drainage, joint and soft tissue mobilization. |  |  |  |  |  |
| For patients with severe ankle sprains, physiotherapists should implement rehabilitation programs that include therapeutic exercises. |  |  |  |  |  |
| When evaluating the results of the rehabilitation program for an ankle sprain, physiotherapists should plan a follow-up until one year since the trauma. |  |  |  |  |  |
