# Supplementary material for: Physiotherapists’ knowledge of and adherence to evidence-based practice guidelines and recommendations for ankle sprains management: a cross-sectional study
Source: BMC Musculoskelet Disord. 2022 Nov 11;23:975. doi: 10.1186/s12891-022-05914-5 (PMC9650827; doi:10.1186/s12891-022-05914-5)
Supplement: Supplementary file 2 — Additional file 2. [file 12891_2022_5914_MOESM2_ESM.docx]

**Supplementary File** **B**

**Survey in Italian language**

Physiotherapists’ Knowledge of and Adherence to Evidence-Based Practice Guidelines and Recommendations for Ankle Sprains Management: a Cross-Sectional Study

***Sezione I: caratteristiche demografiche dei partecipanti***

Gentile Partecipante, Le è stato chiesto di prendere parte ad uno studio dal titolo “Physiotherapists’ Knowledge of and Adherence to Evidence-Based Practice Guidelines and Recommendations for Ankle Sprains Management: a Cross-Sectional Study”.

Prima che Lei prenda una decisione in merito, è importante che comprenda il motivo dello studio e cosa Le sarà chiesto di fare, qualora decidesse di prendervi parte. Lo sperimentatore e i suoi collaboratori sono a Sua completa disposizione per qualsiasi chiarimento. Le prime due sezioni di questo questionario hanno lo scopo di fornirLe un’informazione corretta e completa affinché Lei possa esprimere una scelta libera e consapevole. Il responsabile dello studio è Marco Testa, Professore Aggregato presso il Dipartimento di Neuroscienze, Riabilitazione, Oftalmologia, Genetica e Scienze Materno-Infantili dell'Università di Genova.

La ringraziamo per il Suo prezioso contributo, per qualsiasi domanda può contattarci per e-mail all’indirizzo giuliacaffini95@gmail.com

**A. Nota informativa e consenso informato**

1. Il/la sottoscritto/a dichiara di avere preso visione della "Nota informativa sullo studio" al seguente link bit.ly/2QEBCbv : Sì / NO

2. Il/la sottoscritto/a ha preso visione dell' "Informativa per il trattamento dei dati personali (ex artt. 9 e 10 del Reg. UE n. 2016/679)" al link bit.ly/2RXmSEX e dichiara di PRESTARE IL CONSENSO affinché l’Università degli Studi di Genova tratti i Suoi dati per le finalità e secondo le modalità ivi descritte: Sì / NO

**B. Raccolta dati demografici dei partecipanti**

La preghiamo di rispondere alle seguenti domande:

3. Ha conseguito la Laurea in Fisioterapia in Italia ed esercita attualmente la professione di Fisioterapista in Italia? Sì / NO

4. Le è capitato di aver trattato almeno un paziente con distorsione di caviglia nei precedenti due anni? Sì / NO

5. Età in numero (es. 38): _____

6. Genere con il quale si identifica: maschile / femminile / altro*

*7. Specifichi con quale genere si identifica: ______

8. Anni di lavoro dalla laurea:

- Da meno di 1 anno
- Da 1 a 5 anni
- Da 6 a 10 anni

9. Contrassegni il titolo del percorso accademico che ha conseguito fino ad oggi (più opzioni disponibili):

- Laurea triennale (BSc)
- Post-Graduate I Level Degree
- Master of Science (MSc) / Post-Graduate II Level Degree
- Doctor of Philosophy (PhD)
- Altro (specificare di seguito)*

*10. Se ha svolto uno più percorsi post lauream lo specifichi di seguito (es. "Master di 1° livello in Fisioterapia Sportiva"): ___________________________________________________________

11. Ha frequentato corsi di formazione specifici sull’argomento di “Riabilitazione di pazienti con distorsioni di caviglia”? Sì / NO

***Sezione II: clinical vignette – adherence investigation***

Di seguito trova due casi clinici. La preghiamo di leggerli attentamente e di scegliere quali sono le procedure che metterebbe in atto per la gestione del paziente in prima settimana.

12. Vignette 1

Clinical scenario 1: first episode of acute lateral ankle sprain with negative signs and symptoms for suspecting a bone fracture, acute phase.

Anamnesi: A.R. è una signora di 40 anni, impiegata in ufficio postale, con la passione per il giardinaggio. Ieri ha subito un primo episodio di distorsione laterale di caviglia appoggiando il piede in flessione plantare ed inversione mentre era in giardino. È riuscita a rientrare in casa zoppicando. Il giorno successivo al trauma si presenta presso il Fisioterapista camminando con l’aiuto di due canadesi e tenendo il piede sollevato dal suolo.
Esame obiettivo: alla richiesta di appoggiare il piede per terra per provare a camminare per 4 passi la paziente dichiara di avere paura di sentire dolore, riesce comunque a camminare per tutto lo studio senza zoppicare, ma con un dolore nel compartimento laterale di 4 su 10 sulla scala del dolore VAS (Visual Analogue Scale).
Non presenta dolore alla palpazione dei 6 cm posteriori dei malleoli, né alla zona laterale e mediale del mesopiede. Presenta edema lieve ed ematoma nel compartimento antero-laterale della caviglia.

Scelga nel seguente elenco quali sono le procedure che metterebbe in atto per la gestione del paziente di questo scenario in prima settimana (più opzioni disponibili)

- Applicazione solamente di Ghiaccio/Crioterapia
- Applicazione di Ghiaccio/Crioterapia associata a mobilizzazione attiva tollerata
- Compressione
- Elevazione
- Protezione con tutore semi-rigido
- Protezione con tutore per caviglia con lacci (lace-up brace)
- Protezione con bendaggio elastico (kinesiotape)
- Consiglio al paziente di rivolgersi allo specialista di riferimento o di andare in Pronto Soccorso
- Consiglio al paziente di rivolgersi allo specialista di riferimento o di andare in Pronto Soccorso, iniziando nel frattempo il percorso riabilitativo
- Rinvio il paziente al medico per eventuale cura farmacologica
- Consiglio riposo a letto ed immobilizzazione per 2 settimane
- Consiglio utilizzo della laserterapia
- Consiglio utilizzo della diatermia
- Consiglio utilizzo della elettroterapia antalgica
- Consiglio utilizzo della ultrasuonoterapia
- Tecniche manuali di mobilizzazione articolare passiva da sole
- Tecniche manuali di mobilizzazione articolare passiva associate ad altro trattamento attivo
- Esercizi di mobilità attivi
- Esercizi come: step up, squat, salti e resistenza aerobica

13. Vignette 2

Clinical scenario 2: reinjury acute phase lateral ankle sprain with positive signs and symptoms for suspecting a bone fracture.

Anamnesi: G.C. è una giocatrice di basket di 20 anni che studia all’Università. Due giorni fa, durante la partita, ha subito un episodio di distorsione di caviglia appoggiando il piede in flessione plantare ed inversione nel momento dell’atterraggio da un salto. Si tratta del secondo episodio di distorsione di caviglia, il primo era avvenuto 3 anni fa, dopo il quale aveva seguito un percorso di riabilitazione fino a tornare a giocare.

Questa volta ha dovuto interrompere il gioco, è uscita saltellando sul piede opposto, ha applicato immediatamente il ghiaccio e la caviglia si è gonfiata velocemente. Ha provato ad appoggiare il piede per camminare fino allo spogliatoio ma il dolore era troppo forte (VAS 8/10).
Fino ad oggi ha tenuto il piede elevato con ghiaccio e non lo ha appoggiato per camminare, la notte però la caviglia le fa male (VAS 8/10). Si presenta due giorni dopo il trauma presso il Fisioterapista per la prima visita camminando con due canadesi senza appoggiare il piede.

Esame obiettivo: alla richiesta di appoggiare il piede per terra per provare a camminare per 4 passi la paziente riferisce di provare un dolore 8 su 10 sulla scala del dolore VAS (Visual Analogue Scale), alla palpazione dei 6 cm posteriori al malleolo peroneale riferisce un dolore VAS 7/10.

Scelga nel seguente elenco quali sono le procedure che metterebbe in atto per la gestione del paziente di questo scenario in prima settimana (più opzioni disponibili)

- Applicazione solamente di Ghiaccio/Crioterapia
- Applicazione di Ghiaccio/Crioterapia associata a mobilizzazione attiva tollerata
- Compressione
- Elevazione
- Protezione con tutore semi-rigido
- Protezione con tutore per caviglia con lacci (lace-up brace)
- Protezione con bendaggio elastico (kinesiotape)
- Consiglio al paziente di rivolgersi allo specialista di riferimento o di andare in Pronto Soccorso
- Consiglio al paziente di rivolgersi allo specialista di riferimento o di andare in Pronto Soccorso, iniziando nel frattempo il percorso riabilitativo
- Rinvio il paziente al medico per eventuale cura farmacologica
- Consiglio riposo a letto ed immobilizzazione per 2 settimane
- Consiglio utilizzo della laserterapia
- Consiglio utilizzo della diatermia
- Consiglio utilizzo della elettroterapia antalgica
- Consiglio utilizzo della ultrasuonoterapia
- Tecniche manuali di mobilizzazione articolare passiva da sole
- Tecniche manuali di mobilizzazione articolare passiva associate ad altro trattamento attivo
- Esercizi di mobilità attivi
- Esercizi come: step up, squat, salti e resistenza aerobica

***Section III: statements consensus – knowledge investigation***

Le chiediamo di leggere attentamente le seguenti affermazioni e di indicare quanto si trova in accordo con esse scegliendo un valore da 1 (completamente in disaccordo) a 5 (completamente d’accordo).

14. Quanto si trova in accordo con le seguenti affermazioni nell'ambito della valutazione?

|  | 1  Completamente in disaccordo | 2  Parzialmente in disaccordo | 3  Né d'accordo né in disaccordo | 4  Parzialmente d'accordo | 5  Completamente d'accordo |
| --- | --- | --- | --- | --- | --- |
| La valutazione clinica del danno legamentoso a seguito di una distorsione di caviglia dovrebbe essere eseguita nell’arco delle 48 ore dall’evento traumatico. |  |  |  |  |  |
| Se è presente il sospetto di una frattura della caviglia o del piede, non è raccomandato applicare le Ottawa Ankle Rule. |  |  |  |  |  |
| In anamnesi è importante identificare la presenza di precedenti eventi di distorsione di caviglia. |  |  |  |  |  |
| In presenza di un secondo episodio di distorsione laterale di caviglia non è mai necessario applicare le Ottawa Ankle Rule. |  |  |  |  |  |
| Nel momento della valutazione dei pazienti con distorsione di caviglia, i fisioterapisti dovrebbero utilizzare misure di outcome funzionali come la FAAM (Foot and Ankle Ability Measure). |  |  |  |  |  |

15. Quanto si trova in accordo con le seguenti affermazioni nell'ambito del trattamento?

|  | 1  Completamente in disaccordo | 2  Parzialmente in disaccordo | 3  Né d'accordo né in disaccordo | 4  Parzialmente d'accordo | 5  Completamente d'accordo |
| --- | --- | --- | --- | --- | --- |
| In presenza di distorsioni di caviglia ricorrenti si dovrebbe consigliare al paziente di eseguire un programma di esercizio terapeutico per la coordinazione e l’equilibrio per almeno 1 anno dal trauma. |  |  |  |  |  |
| Il tutore ha il ruolo di prevenire eventi recidivanti di distorsioni di caviglia laterale. |  |  |  |  |  |
| Almeno una delle seguenti modalità di trattamento è fortemente raccomandata nella gestione del paziente con distorsione di caviglia in fase acuta: ultrasuonoterapia, laserterapia, elettroterapia antalgica, diatermia. |  |  |  |  |  |
| In individui con distorsione di caviglia, il Fisioterapista dovrebbe usare tecniche di terapia manuale come linfodrenaggio, mobilizzazione articolare e dei tessuti molli. |  |  |  |  |  |
| In presenza di una distorsione di caviglia severa, il fisioterapista dovrebbe sviluppare un programma di riabilitazione che includa l’esercizio terapeutico. |  |  |  |  |  |
