# Supplementary material for: Physiotherapists’ knowledge of and adherence to evidence-based practice guidelines and recommendations for ankle sprains management: a cross-sectional study
Source: BMC Musculoskelet Disord. 2022 Nov 11;23:975. doi: 10.1186/s12891-022-05914-5 (PMC9650827; doi:10.1186/s12891-022-05914-5)
Supplement: Supplementary file 3 — Additional file 3. [file 12891_2022_5914_MOESM3_ESM.docx]

**Supplementary File** **C**

**Evidence-Based Practice recommendations**

Physiotherapists’ Knowledge of and Adherence to Evidence-Based Practice Guidelines and Recommendations for Ankle Sprains Management: a Cross-Sectional Study

***Section II: clinical vignette – adherence investigation***

| **Vignette 1**: first episode of acute LAS with negative signs and symptoms for suspecting a bone fracture (negative Ottawa ankle rules). | |
| --- | --- |
| **Proposed treatments** | **EBP Recommendations** |
| Application of ice/  cryotherapy alone | **CS 2018**: The individual aspects of RICE are not effective, apart from cryotherapy, if provided in combination with exercise therapy. There is no evidence that RICE alone, or cryotherapy, or compression therapy alone has any positive influence on pain, swelling or patient function. Therefore, there is no role for RICE alone in the treatment of acute LAS (level 2).  **CPGs 2021**: Clinicians may use repeated intermittent applications of ice in association with a therapeutic exercise program to address symptoms and functioning following an acute LAS. (GRADE C) |
| Application of ice/ cryotherapy in combination with tolerated active mobilization | **CPGs 2013**: Clinicians should use repeated intermittent applications of ice to reduce pain, decrease the need for pain medication, and improve weight bearing fol­lowing an acute ankle sprain. (GRADE A)  **CS 2018**: The individual aspects of RICE are not effective, apart from cryotherapy, if provided in combination with exercise therapy. (level 2).  **CPGs 2021**: Clinicians may use repeated intermittent applications of ice in association with a therapeutic exercise program to address symptoms and functioning following an acute LAS. (GRADE C) |
| Compression | **CS 2018**: There is no evidence that RICE alone, or cryotherapy, or compression therapy alone has any positive influence on pain, swelling or patient function. Therefore, there is no role for RICE alone in the treatment of acute LAS (level 2). |
| Elevation | **CS 2018**: There is no evidence that RICE alone, or cryotherapy, or compression therapy alone has any positive influence on pain, swelling or patient function. Therefore, there is no role for RICE alone in the treatment of acute LAS (level 2). |
| Protection with a semi-rigid brace | **CPGs 2013**: Clinicians should advise patients with acute lat­eral ankle sprains to use external supports and to progressively bear weight on the affected limb. (Grade A)  **CS 2018**: Use of functional support for 4–6 weeks is preferred over immobilisation. The use of an ankle brace shows the greatest effects compared with other types of functional support (level 2).  **CPGs 2021**: Clinicians should advise patients with an acute LAS to use external supports, such as braces or taping, and to progressively bear weight on the affected limb. The type of external support and gait assistive device recommended should be based on the severity of the injury, phase of tissue healing, level of protection indicated, extent of pain, and patient preference. (GRADE A) |
| Protection with a lace-up brace | As above |
| Protection with elastic tape (kinesiotape) | As above |
| Advice to the patient to contact the specialist or to go to the emergency room | **CPGs 2013**: The Ottawa and Bernese ankle rules should be used to determine whether a radiograph is required to rule out a fracture of the ankle and/or foot.  **CPGs 2021**: Clinicians should conduct a thorough patient history and examine the multiple segments of the ankle-foot complex to rule in or out the pathologies that may be present when differentially diagnosing an acute sprain and utilize the OAR when determining whether a radiograph is necessary after an acute LAS. |
| Advice to the patient to contact the specialist or to go to the emergency room, starting in the meantime the rehabilitation program | **CPGs 2013**: The Ottawa and Bernese ankle rules should be used to determine whether a radiograph is required to rule out a fracture of the ankle and/or foot.  **CPGs 2021**: Clinicians should conduct a thorough patient history and examine the multiple segments of the ankle-foot complex to rule in or out the pathologies that may be present when differentially diagnosing an acute sprain and utilize the OAR when determining whether a radiograph is necessary after an acute LAS. |
| Referral of the patient to the doctor for a possible pharmacological treatment | **CS 2018**: NSAIDs may be used by patients who have incurred an acute LAS for the primary purpose of reducing pain and swelling. However, care should be taken in NSAID usage as it is associated with complications (level 2) and may suppress or delay the natural healing process.  **CPGs 2021**: Clinicians may prescribe NSAIDs (as physical ther­apy practice acts allow) to reduce pain and swelling in those with an acute LAS. (GRADE C) |
| Advice to rest and immobilization for 2 weeks | **CPGs 2013**: there was a significant benefit to weight bearing as tolerated compared to non–weight-bearing cast immobilization. (Level I) Clinicians should advise patients with acute lat­eral ankle sprains to use external supports and to progressively bear weight on the affected limb. (Grade A)  **CS 2018**: Use of functional support and exercise therapy is preferred as it provides better outcomes compared with immobilisation. If immobilisation is applied to treat pain or oedema, it should be for a maximum of 10 days after which functional treatment should be commenced (level 2). |
| Recommend for laser therapy | **CPGs 2013**: There is moderate evidence both for and against the use of low-level laser therapy for the manage­ment of acute ankle sprains. (GRADE D)  **CS 2018**: As no strong evidence exists on the effectiveness of these treatment modalities, they are not advised in the treatment of acute LAS (level 2).  **CPGs 2021**: Clinicians may use low-level laser therapy to reduce pain in the initial phase of an acute LAS. (GRADE C) |
| Recommend for diathermy  endurance | **CPGs 2013**: Clinicians can utilize pulsating shortwave diather­my for reducing oedema and gait deviations associ­ated with acute ankle sprains. (GRADE C)  **CS 2018**: As no strong evidence exists on the effectiveness of these treatment modalities, they are not advised in the treatment of acute LAS (level 2).  **CPGs 2021**: Not changed from 2013 |
| Recommend for antalgic electrothera-py | **CPGs 2013**: There is moderate evidence both for and against the use of electrotherapy for the management of acute ankle sprains. (GRADE D)  **CS 2018**: As no strong evidence exists on the effectiveness of these treatment modalities, they are not advised in the treatment of acute LAS (level 2).  **CPGs 2021**: Not changed from 2013 |
| Recommend for ultrasound therapy | **CPGs 2013**: Clinicians should not use ultrasound for the man­agement of acute ankle sprains. (GRADE A)  **CS 2018**: As no strong evidence exists on the effectiveness of these treatment modalities, they are not advised in the treatment of acute LAS (level 2).  **CPGs 2021**: Not changed from 2013 |
| Passive joint mobilization with manual therapy techniques alone | **CPGs 2013**: Clinicians should use manual therapy procedures, such as lymphatic drainage, active and passive soft tis­sue and joint mobilization, and anterior-to-posterior talar mobilization procedures, within pain-free movement to reduce swelling, improve pain-free ankle and foot mobility, and normalize gait parameters in individuals with an acute lateral ankle sprain. (GRADE B)  **CPGs 2021**: Clinicians should use manual therapy procedures, such as lymphatic drainage, active and passive soft tissue and joint mobilization, and anterior-to-posterior talar mobilization procedures within pain-free movement, **alongside therapeutic exercise** to reduce swelling, improve pain-free ankle and foot mobility, and normalize gait parameters in individuals with a LAS. (GRADE A) |
| Passive joint mobilization with manual therapy techniques in combination with other active treatments | **CPGs 2013**: Clinicians should include manual therapy procedures, such as graded joint mobilizations, manipulations, and non–weight-bearing and weight-bearing mobilization with movement, to improve ankle dorsiflexion, proprioception, and weight-bearing tol­erance in patients recovering from a lateral ankle sprain. (GRADE A)  **CS 2018**: A combination with other treatment modalities, such as exercise therapy, enhances the efficacy of manual joint mobilisation and is therefore advised (level 3).  **CPGs 2021**: Clinicians should use manual therapy procedures, such as lymphatic drainage, active and passive soft tissue, and joint mobilization, and anterior-to-posterior talar mobilization procedures within pain-free movement, **alongside therapeutic exercise** to reduce swelling, improve pain-free ankle and foot mobility, and normalize gait parameters in individuals with a LAS. (GRADE A) |
| Active mobility exercises | **CS 2018**: Exercise therapy should be commenced after LAS to optimise recovery of joint functionality. (LEVEL 1) For this reason, it is advised to start exercise therapy, especially in athletes, as soon as possible after the initial sprain to prevent recurrent LAS. Exercise therapy should be included into regular training activities as much as possible as home-based exercise (level 1).  **CPGs 2021**: Clinicians should implement rehabilitation pro­grams with a structured therapeutic exercise pro­gram, which can include protected active ROM, stretching exercises, neuromuscular training, postural re-ed­ucation and balance training, both in clinic and at home, as determined by injury severity, identified impairments, prefer­ences, learning needs, and social barriers in those with a LAS. (GRADE A) |
| Exercises such as step up, squat, jumps and aerobic exercises | **CS 2018**: Supervised exercises focusing on a variety of exercises such as proprioception, strength, coordination, and function will lead to a faster return to sport in patients after a LAS and are therefore recommended (level 1).  **CPGs 2021**: Clinicians should implement rehabilitation pro­grams with a structured therapeutic exercise pro­gram, which can include protected active ROM, stretching exercises, neuromuscular training, postural re-ed­ucation and balance training, both in clinic and at home, as determined by injury severity, identified impairments, prefer­ences, learning needs, and social barriers in those with a LAS. (GRADE A) |

| **Vignette 2**: reinjury acute lateral ankle sprain with positive signs and symptoms for suspecting a bone fracture (positive Ottawa ankle rules). | |
| --- | --- |
| **Proposed treatments** | **EBP Recommendations and authors’ comments** |
| Advice to the patient to contact the specialist or to go to the emergency room | **CPGs 2013**: the Ottawa and Bernese ankle rules should be used to determine whether a radiograph is required to rule out a fracture of the ankle and/or foot. (GRADE A)  **CPGs 2021**: Clinicians should conduct a thorough patient history and examine the multiple segments of the ankle-foot complex to rule in or out the pathologies that may be present when differentially diagnosing an acute sprain and utilize the OAR when determining whether a radiograph is necessary after an acute LAS.  **Authors’ comment:** considered FOLLOWING if chosen alone; considered PARTIALLY FOLLOWING if chosen with RICE components or brace components. According to the text where the authors explicated that the Ottawa ankle rules are positive (there is pain in the malleolar zone and there is tenderness along the tip of the posterior edge of the distal 6 cm of the lateral malleo­lus, and inability to bear weight for 4 steps), it’s an emergency context, therefore the physiotherapists should rule out a bone fracture before providing any treatments.  Anytime the choice of the participants was missing the referral to the doctor or to the emergency room, it was considered NOT FOLLOWING. |
| Advice to the patient to contact the specialist or to go to the emergency room, starting in the meantime the rehabilitation program | As above.  **Authors’ comment:** considered NOT FOLLOWING anytime. According to the text where the authors explicated that the Ottawa ankle rules are positive, it’s an emergency context, therefore the physiotherapists should rule out a bone fracture before providing any treatments. |
| **Legend**: CPGs 2013= Clinical Practice Guidelines from Martin et al 2013; CS 2018 = Consensus Statement from Vuuberg G. et al 2018; CPGs 2021 = Clinical Practice Guidelines from Martin et al 2021. | |

***Section III: statements consensus – knowledge investigation***

| **Table 2** Section III: Statements and review of EBP recommendations | |
| --- | --- |
| **Statements about assessment** | **EBP recommendations** |
| 1) The clinical assessment of damage to the ligaments after an ankle sprain should be performed within 24 hours from the trauma. (**Reversed statement**) | **CS 2018**: Regarding the clinical assessment of damage to the anterior talofibular ligament, the sensitivity (84%) and specificity (96%) of assessment using the anterior drawer are optimised if clinical assessment is delayed for between 4 and 5 days post injury. (level 2) |
| 2) In case of suspected fracture of the ankle or the foot, it’s not recommended to apply the Ottawa ankle rules. (**Reversed statement**) | **CS 2018**: In case of a suspected fracture, the OAR should be applied (level 2).  **CPGs 2021**: Clinicians should conduct a thorough patient history and examine the multiple segments of the ankle-foot complex to rule in or out the pathologies that may be present when differentially diagnosing an acute sprain, and utilize the OAR when determining whether a radiograph is necessary after an acute LAS. |
| 3) During the anamnesis it is important to assess previous events of ankle sprains. | **CPGs 2013**: Clinicians should recognize the increased risk of acute lateral ankle sprain in individuals who have a history of a previous ankle sprain. (GRADE B)  **CPGs 2021**: Clinicians should include patient age, BMI, pain coping strategies, report of instability, history of previous sprain, ability to bear weight, pain with weight bearing, ankle dorsiflexion ROM, medial joint-line tenderness, balance, and ability to jump and land (as safely tolerated) in their initial assessment because of their role in influencing the clinical course and estimation of time to ac­complish the goals of an individual with an acute LAS. (GRADE B) |
| 4) In front of a second episode of lateral ankle sprain it is never necessary to apply the Ottawa ankle rules. (**Reversed statement**) | **CPGs 2013**: the Ottawa and Bernese ankle rules should be used to determine whether a radiograph is required to rule out a fracture of the ankle and/or foot. (GRADE A)  **CS 2018**: In case of a suspected fracture, the OAR should be applied (level 2) |
| 5) Physiotherapists should incorporate functional outcome measures such as the FAAM (Foot and Ankle Ability Measure), as part of the examination of patients with ankle sprain. | **CPGs 2013**: Clinicians may incorporate a discriminative instru­ment, such as the Cumberland Ankle Instability Tool, to assist in identifying the presence and se­verity of ankle instability. (GRADE B)  **CPGs 2013**: Clinicians should incorporate validated function­al outcome measures, such as the FAAM and the LEFS, as part of a standard clinical examination. (GRADE A)  **CPGs 2021**: Clinicians should use validated patient-reported outcome measures, such as the PROMIS PF and PI scales, the FAAM, and the LEFS, as part of a stan­dard clinical examination. (GRADE A) |
| **Statements about treatment** | **EBP recommendations** |
| 6) When evaluating the results of the rehabilitation programme for an ankle sprain, physiotherapists should plan a follow-up until one year since the trauma. | **CPGs 2013**: 44% of subjects had persistent symptoms at 1-year follow-up, 5% to 33% of patients continued to have pain at 1-year or longer follow-up, with 5% to 25% still experiencing pain after 3 years. |
| 7) The brace has a role in the prevention of recurrent lateral ankle sprains events. | **CS 2018**: Both tape and brace have a role in the prevention of recurrent LAS despite limited evidence on mechanisms that leads to these beneficial effects (level 1). The choice of usage should depend on personal preferences.  **CPGs 2021**: Clinicians should recommend the use of prophylactic bracing to reduce the risk of a first-time LAS, particularly for those with risk factors for LAS. |
| 8) At list one of the following treatment modalities is strongly recommended for the management of patients with ankle sprain during the acute phase: ultrasound, laser therapy, electrotherapy, diathermy. (**Reversed statement**) | **CPGs 2013**:  *Low-level laser therapy*: there is moderate evidence both for and against the use of low-level laser therapy for the management of acute ankle sprains. (D)  *Electrotherapy*: there is moderate evidence both for and against the use of electrotherapy for the management of acute ankle sprains. (GRADE D)  *Diathermy*: clinicians can utilize pulsating shortwave diathermy for reducing oedema and gait deviations associated with acute ankle sprains. (GRADE C)  *Ultrasound*: clinicians should not use ultrasound for the man­agement of acute ankle sprains. (GRADE A)  **CS 2018**: As no strong evidence exists on the effectiveness of these treatment modalities, they are not advised in the treatment of acute LAS (level 2).  **CPGs 2021**: not changed. |
| 9) In the treatment of patients with an ankle sprain, clinicians should use manual therapy procedures, such as lymphatic drainage, joint and soft tissue mobilization. | **CPGs 2013**: Clinicians should use manual therapy procedures, such as lymphatic drainage, active and passive soft tissue and joint mobilization, and anterior-to-pos­terior talar mobilization procedures, within pain-free move­ment to reduce swelling, improve pain-free ankle and foot mobility, and normalize gait parameters in individuals with an acute lateral ankle sprain. (GRADE B) |
| 10) For patients with severe ankle sprains, physiotherapists should implement rehabilitation programs that include therapeutic exercises. | **CPGs 2013**: Clinicians should implement rehabilitation programs that include therapeutic exercises for patients  with severe lateral ankle sprains. (GRADE A) |
| 11) When evaluating the results of the rehabilitation programme for an ankle sprain, physiotherapists should plan a follow-up until one year since the trauma. | **CPGs 2013**: 44% of subjects had persistent symptoms at 1-year follow-up, 5% to 33% of patients continued to have pain at 1-year or longer follow-up, with 5% to 25% still experiencing  pain after 3 years. |
| **Legend**: CPGs 2013= Clinical Practice Guidelines from Martin et al 2013; CS 2018 = Consensus Statement from Vuuberg G. et al 2018; CPGs 2021 = Clinical Practice Guidelines from Martin et al 2021; reversed statement = the expected answer is 1-2 on a 5-point Likert scale | |
